# A comprehensive interventional program for promoting father’s participation in the perinatal care: protocol for a mixed methods study

**DOI:** 10.1186/s12978-018-0572-x

**Published:** 2018-08-25

**Authors:** Vahideh Firouzan, Mahnaz Noroozi, Ziba Farajzadegan, Mojgan Mirghafourvand

**Affiliations:** 1 Student Research Center, Faculty of Nursing and Midwifery, Isfahan University of Medical Sciences, Isfahan, Iran; 2 Department of Midwifery and Reproductive Health, School of Nursing and Midwifery, Isfahan University of Medical Sciences, Isfahan, Iran; 3 Department of Community Medicine, Medicine School, Isfahan University of Medical Sciences, Isfahan, Iran; 4 Social Determinants of Health Research Center, Tabriz University of Medical Sciences, Tabriz, Iran

**Keywords:** Father, Participation, Perinatal care, Iran

## Abstract

**Background:**

The inclusion of fathers’ participation during the perinatal period is an important strategy for improving mothers’ health. No studies have yet been conducted in Iran to explain the concept, obstacles and facilitators of fathers’ participation during the perinatal period. Thus, this study will be carried out to provide a comprehensive interventional program for promoting fathers’ participation in the perinatal care.

**Methods:**

This study is a sequential exploratory (qualitative – quantitative) mixed methods design that consists of three consecutive phases. In this study, following a qualitative approach, the researchers will explain the concept, obstacles, facilitators and strategies related to promoting fathers’ participation in perinatal care. In the second phase, researchers will design an appropriate and comprehensive interventional program for promoting fathers’ participation in perinatal care by using the results of the qualitative phase and literature reviews. The proposed interventional program is designed by a panel of experts based on prioritization guidelines and will be finalized for execution. In the third stage, the effectiveness of interventional program on the awareness, attitude and practice of fathers about participation in perinatal care will be investigated in a semi-experimental study.

**Discussion:**

It is expected that from the results of the present mixed methods study, by presenting an interventional culturally sensitive program which is appropriate for the conditions of the society for expectant fathers, the participation of fathers in the perinatal period will increase and thus lead to improvements in the health of the mother and the infant. If this interventional program is effective, it could be included in the perinatal health care guidelines.

**Trial registration:**

IRCT20160224026756N4 Registered 27 May 2018.

## Plain English summary

Fathers’ participation during the perinatal period is an important strategy for improving mothers’ health**.** The results of this study offer a rich source of information for the required interventions to promote the health of the mothers and infants during pregnancy and postpartum period. This study is a sequential exploratory (qualitative – quantitative) mixed methods design that consists of three consecutive phases. In this study, following a qualitative approach, the researchers will explain the concept, obstacles, facilitators and strategies related to promoting fathers’ participation in perinatal care. In the second phase, the researcher will design an appropriate interventional program for promoting fathers’ participation in perinatal care by using the results of the qualitative phase and literature reviews. The purposed interventional program is designed by a panel of experts based on prioritization guidelines and will be finalized for execution. In the third stage, the effectiveness of interventional program on the awareness, attitude and practice of fathers about participation in perinatal care will be investigated in a semi-experimental study. Therefore, it is expected that conducting a mixed method study by presenting an interventional culturally sensitive program which is appropriate for the conditions of the society for expectant fathers, the participation of fathers in the perinatal period will increase and thus, improve the health of the mother and the infant.

## Background

Throughout life, special stages which would leave a profound impact on individual’s life exist [1]. One of these stages is pregnancy and delivery, which is the greatest event in a woman’s life and her family. According to the 7th clinical guideline of the American College of Obstetricians and Gynecologists, and the American Academy of Pediatrics in 2012, perinatal care include a full range of preconception counseling, preparing prenatal and delivery care, neonatal care and postpartum care [2] and the main goal of these care is to prevent any complications for the mother and the infant [3]. Men’s participation is an important strategy for reaching the third Millennium Development Goals (MDGs) such as women’s empowerment and improvement of mothers’ health [4]. Many studies have mentioned the positive outcomes of men’s participation during pregnancy and delivery [5–8]. In this regard, in the International Conference on Population and Development (ICPD) in 1994, the shared responsibility of men and improvement of their active participation in parental responsibilities, sexual behaviors and reproductive responsibility in family planning, pregnancy health, maternal-neonatal health, preventing unwanted and risky pregnancies, health and nutrition of the children and preventing violence against women and children, were emphasized [9]. But despite this emphasis and international agreement on the importance of men’s participation for the improvement of maternal and neonatal health, advancements in this regard are slow and there are challenges in preparing the environments for the presence and participation of men in the care of mothers [10], in a way that the United Nations Population Fund in 2009 stated that:“ Despite the significant evidence about the benefits of men’s participation thus far, and although men have responded well to the efforts of their participation during delivery., their participation during pregnancy, delivery and childcare has not been effectively promoted globally” [9].

No specific formula exists for men’s participation in the healthcare system and the most accepted strategies are those that are culturally acceptable, appropriate and applicable. To find these strategies, research and need-assessment should be conducted [11]. In this regard, during the recent years in industrial Western countries, fathers’ participation and presence during pregnancy and delivery has been increased [12]; but in most parts of the world, men’s participation is the missing link in countries’ and governments’ policies, information and plans and it is usually neglected and most of the global health systems have no specific plan for attracting men’s participation in reproductive health programs [13]. In Iran, like many other countries, the governmental departments have considered the usual care for women, but no comprehensive plan exists for men. Only during recent years, for encouraging women to have vaginal delivery, delivery preparation classes are being conducted for pregnant women in 8 sessions and the husband can participate in one session. But practically, this matter is just being executed at a limited number of health centers and there are problems for executing this program. Also, in some hospitals, fathers who have participated in the delivery preparation classes are allowed to be with their wives during labor and delivery but this is also very limited. Furthermore, the paternity leave for fathers, which is a two-week leave, would only be given to men with working wives; but this could not be generalized for all of the male employees or even to those whose wives have a part-time job with no insurance policy [14]. However, this plan is still not being implemented by all the organizations and offices. So far, most of the conducted studies about men’s participation, internationally and nationally, were about family planning, sexually transmitted diseases (STD) and AIDS; while other aspects of women’s health like pregnancy and delivery were not considered much; even though the need for information in these fields is necessary [12].

Therefore, regarding the informational challenge about father’s role in mothers’ health and also due to the lack of a comprehensive program for father’s participation based on Iranian culture, this study will be carried out to provide a comprehensive and culturally sensitive program for father’s participation during the perinatal period.

### Objectives

The objectives of each phase are as follows:

#### Objectives of the first phase: qualitative study

To explain the concept, obstacles, facilitators and strategies of father’s participation in perinatal care.

#### Objectives of the second phase: program design

To design a preliminary interventional program based on the data extracted from the qualitative phase and the review of texts.

To validate the interventional program by a panel of experts.

#### Objectives of the third phase: quantitative study

To determine the effect of the designed interventional program on awareness, attitude and practice of fathers about participation in perinatal care.

## Methods/Design

**Fig. 1:**
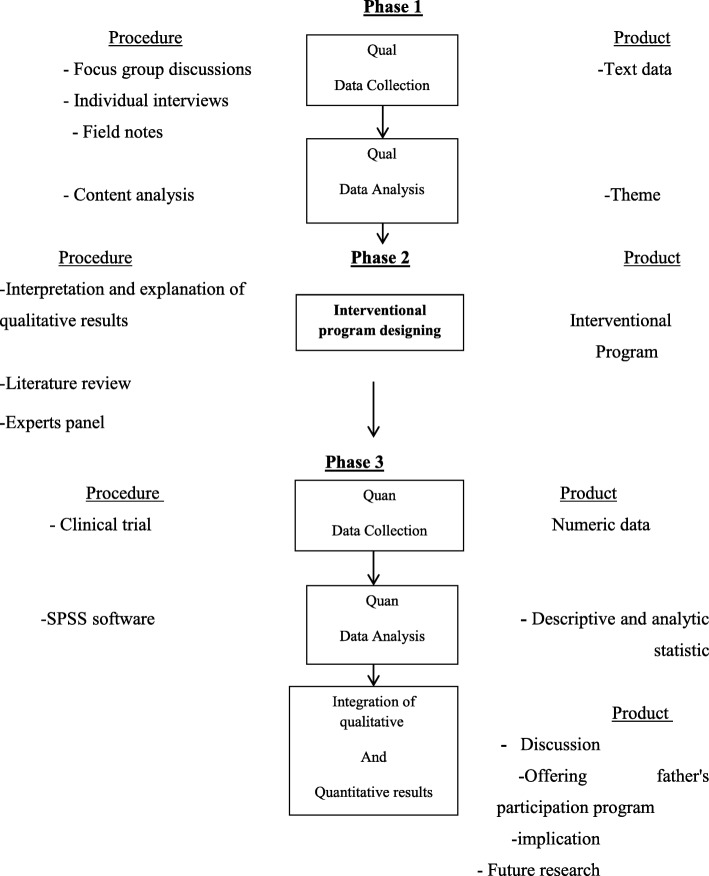
Study visual diagram

This study is a sequential exploratory (qualitative – quantitative) mixed methods design that consists of three consecutive phases. In this study, following a qualitative approach, the researchers will explain the concept, obstacles, facilitators and strategies related to promoting father’s participation in perinatal care**.** In the second phase, the researcher will design an appropriate interventional program for promoting father’s participation in perinatal care by using the results of the qualitative phase and literature reviews (Fig. 1). The purposed intervention is designed by a panel of experts based on prioritization guidelines and will be finalized for execution. In the third stage, the effectiveness of interventional program on the awareness, attitude and practice of fathers on their participation in perinatal care will be investigated in a semi-experimental study.

### Phase I: Qualitative study

At this phase, the researcher is trying to discover the concept and identification of barriers and strategies for father’s participation in the perinatal period to design an interventional program for their participation. This study is carried out using qualitative content analysis method.

#### Participants in the qualitative phase

Participants of this phase will be men whose wives are pregnant or have experienced delivery, pregnant or delivered women, midwives, gynecologists and nurses, and also managers at the level of health deputy of the Tabriz University of Medical Sciences, Iran as well as maternal health policy makers of the Ministry of Health and Medical Education.

#### Sampling method

In the qualitative phase of this study, the sampling will start purposively [15] and continue considering the maximum variety in educational level, socioeconomic condition, job, age, and number of pregnancies and deliveries (for the mothers group). Also, healthcare providers with different working experience and from different health centers as well as maternal health policy makers would be recruited for the study.

#### Inclusion criteria for men and women

The inclusion criteria are willingness to participate in the study, providing informed consent, being able to understand and express their experiences and having Iranian Nationality.

#### Inclusion criteria for care providers

Having at least 5 years of working experience and willingness to participate in the study.

#### Research environment

The interviews will be conducted at the time and place of the participants’ desire (hospitals, midwifery or gynecologists’ offices, prenatal clinics, universities, home, etc.).

#### Data collection process

After selecting each participant, the researcher will introduce herself and state the main objectives of the study in order to gain participant’s trust to ensure the confidentiality of the interviews. The researchers will ensure that the participants have their freedom to discontinue their cooperation with the study whenever they want. After obtaining a verbal and written consent from the participants, the interviews will be recorded using MP4 device. In case where sound recording is not allowed, interviews will be conducted by taking notes.

Data will be collected through in-depth interviews, focus group discussions and field notes. The interviews will begin with the open questions of “How men can participate in the care of their wives during pregnancy, delivery and after delivery? What are the men’s participation barriers in perinatal care and what are the men’s participation facilitators in perinatal care? Please explain.” And then the paths of the interviews will be guided through participants’ open and interpretative answers. Data gathering will continue until data saturation [16] and no new data code will emerge in the interviews.

#### Data analysis

Data will be analyzed using conventional content analysis [17]. At the first opportunity after recording the interviews, the researcher will regularly change the recordings into texts. Then the written texts will be reviewed repeatedly to achieve a comprehensive understanding of the interviews. Afterward, sentences and phrases will be coded and after creating the codes using inductive method; similar codes will be merged with each other and those with similar meanings will be put in the same categories to create the sub-categories. This will be done by constant comparison of all the data. Eventually, using inductive process, similar sub-categories will be merged into main categories.

#### Accuracy and reliability of qualitative data

To judge the correctness of the data, dependability, credibility, transferability and confirmability standards will be used [15]. To provide the credibility of the results, different methods including in-depth interviews at different times and places, a combination of data gathering methods such as individual interviews, focus group discussions and the maximum variation in selecting the participants will be used. Review by the participants will be used to confirm the accuracy of the data and extracted codes; also, experts’ opinions will be used to ensure that the results are compatible with participants’ opinions. In the present study, for increase transferability, the results will be given to people who did not participate in the study and had similar characteristics to the participants so that they would judge about the similarity of the results based on their own experiences.

### Phase II: Designing interventional program

After explaining the concept, barriers and facilitators of father’s participation in perinatal care and also determining the interventional strategies from the participants’ point of view in the qualitative study, the researcher will review related papers and texts. At this stage, the search and review of the relevant texts will be done to confirm and complete the interventional strategies taken in the qualitative phase of the study thereby designing an interventional program for father’s participation in perinatal care.

The method of review will be narrative review with searching in electronic and library resources including reference books and theses. Databases that will be used to search and identify related articles, are PubMed, Cochrane, Google, Google scholar, SID, Magiran, Iranmedx, Scopus, Science Direct, Uptodate, Springer, Biomed Central, WHO and UNFPA. All the studies published between 2005 and 2018, both in English and Persian languages with qualitative, quantitative, and mixed methods study and with the keywords: male involvement, men’s participation**,** fathers’ participation, fathers’ involvement, reproductive health, perinatal care, maternity care, perinatal period, maternal and newborn health, safe motherhood, safe delivery, barriers, intervention, interventional strategy, antenatal and postnatal care, prenatal care, pregnancy and childbirth, paternal support, program, postpartum care, will be reviewed. To perform the search, these keywords including And or Space Operators between different words and from Or to similar words will be used. The star’s mark will be used at the end of the phrase to find the exact phrase of the string and to search for all of the items.

#### Holding a panel of experts

At this stage, the drafting of the strategies extracted from the qualitative research and review of the text will be prioritized using the decision matrix. In this way, these strategies will be presented to a number of experts in the first Delphi round in terms of cost, ease of implementation, timing and effectiveness; and for each dimension, a score between 1 and 3 will be considered.

After completing the decision matrix by them and collecting comments, prioritizing of strategies based on the score given by each member will be done for each dimension of intervention and strategies related to each dimension. Then, an intervention program will be designed for the highest priority strategies and in the second round of Delphi, a preliminary version of the proposed intervention program will be designed, and will be presented and evaluated in panel discussion in the presence of the research team and experts (including reproductive health specialists, gynecologists, midwives and managers at the level of health deputy). A few days before the meeting, a copy of the proposed intervention program will be made available to the panel members to write their comments. Then, based on the panel members’ views, corrections will be made to the designed intervention program, and subsequently finalized and implemented in the quantitative phase (Phase III of the study).

### Phase III: Quantitative study

#### Type and direction of the quantitative study

The quantitative phase of the research will be implemented as a three-group semi-experimental study.

#### The studied population

In this study, the target population is all pregnant women with a gestational age of 20 to 22 weeks and whose husbands have referred to prenatal clinics affiliated to the Social Security Organization of Tabriz.

#### Research sample

The research sample will be formed from the study population who entered the study with convenience sampling and have the inclusion criteria.

#### Research environment

The research environment is three prenatal clinics which are affiliated to the Social Security Organization of Tabriz city. The reason for choosing such environment is the convenient access to pregnant women and their husbands in these settings and due to the fact that these centers have high daily attendance rates.

#### Sample size

The sample size calculation is based on the results of previous studies, based on knowledge, attitude and practice variables using G-power software. The maximum sample size based on the practice variable and considering the data [power 95%, two sided α = 0.05, sd_2_ = 6.34, m_2_ = 88.81, sd_1_ = 14.69, m_1_ = 76.18] will be calculated with 22 people in each group, and due to probability of 10% loss, total sample size will be considered as 24. The final sample size will be determined based on the pilot study.

#### Sampling method

This clinical trial has two intervention groups and one control group; each will be randomly (lottery) selected from a prenatal clinic and then samples will be selected based on convenience sampling method in clinics.

#### The inclusion criteria

The inclusion criteria for this phase will include; being literate; not having passed any educational courses about participation in perinatal care; living harmoniously with spouse and without any challenge between couples.

#### Exclusion criteria

The exclusion criteria will include unwillingness to continue cooperation at each stage of the research and failure to receive 50% of the intervention for any reason.

#### Study variables

In this study, the designed interventions will be considered as an independent variable, while awareness, attitude and practice of fathers about participation in perinatal care will be considered as dependent variables.

#### Data collection

The tools used in the quantitative phase of this research are researcher-made questionnaires. The first part consists of demographic and reproductive characteristics; while the second part of the questionnaire is to assess the awareness, attitude and practice of fathers which will be completed before the intervention and 3 months after the intervention. The knowledge and attitude questionnaires will be completed by fathers and father’s practice questionnaire will be completed by their wives. The awareness questionnaire consists of 28 questions, which have three options: “correct”, “false” and “not sure.” The attitude questionnaire, which consists of 23 questions, is based on the Likert scale of 5 options with the answer “I totally agree”, “agree”, “do not comment”, “disagree”, and “completely disagree”. The practice questionnaire has 23 questions, based on the Likert scale, with the options “Never”, “rarely”, “sometimes”, “most often” and “always”.

#### The implementation method

The researcher will implement the proposed intervention after obtaining a permit from the Ethics Committee of Tabriz University of Medical Sciences and performing the necessary coordination with the designated centers’ officials. At first, through prenatal care records, the list of pregnant women with a gestational age of 20 to 22 weeks will be extracted and through phone, they will be invited together with their husband to participate in the research. After reaching the sample, knowledge and attitude of fathers in the intervention and control groups will be measured by questionnaires before intervention and their practice about participation in prenatal care will be measured by using questionnaire completed by their wives. After the implementation of the intervention program, the questionnaires will be completed again in the intervention and control groups.

#### Data analysis

The collected data will be analyzed using descriptive statistical methods (mean, standard deviation, minimum and maximum) and inferential statistics (One way analysis of variance, paired t-test, Chi-square, Fisher’s exact test, and Mann-Whitney) and by using SPSS 23 software.

#### Integration of the qualitative and quantitative data

The results of the qualitative and quantitative phases of the study will be integrated and finally, a program for father’s participation in perinatal care will be provided.

## Discussion

Considering that fathers’ participation in perinatal care is an important key factor in the improvement of mother’s and infant’s health, therefore, the need for fundamental interventions in this field is essential. In this mixed methods study, sequential exploratory approach will be used for providing an interventional program sensitive to the culture and conditions of the Iranian society for men’s participation in perinatal care. Sequential exploratory approach is a known method for researching, especially when little information is available about the study subject. It is an appropriate method for achieving participants’ experiences. When one method is not sufficient for revealing the study subject, it is better to use a combination of two methods [18]. Using a mixed methods study will help the researcher gain a more comprehensive understanding of the phenomenon and relate its different aspects with each other [19, 20]. Since the present research question has a new multidimensional nature and the researcher is not able to predict the factors that will affect the subject, therefore, it seems that the use of a mixed methods study and the application of both the qualitative and quantitative methods will be an appropriate way to reach the goals of the study. Men’s participation in all of the stages of the mother’s and infant’s life has significant effects on their physical and psychological health; and international conducted studies have mentioned the great potential of fathers for participation during pregnancy, delivery and postpartum periods on decreasing mothers’ mortality and morbidity rates [21]. According to the report of the World Health Organization, 99% of these deaths could be prevented [22] and also, addressing the obstacles for using perinatal care and providing a supportive environment in the society and the family for women’s access to these care have been mentioned as the main strategies for decreasing mothers’ mortality rate [23]. Therefore, it seems that the results of the present study will help policymakers, managers of health deputies, healthcare providers and families especially husbands to perform their essential roles in improving fathers’ participation in perinatal care by using the presented interventional program, and consequently have a positive effect on mothers’ and infants’ health and decrease their rate of mortality and morbidity.

## Abbreviations

AIDS: Acquired Immune Deficiency Syndrome
ICPD: International Conference on Population and Development
MDGs: Millennium Development Goals
STD: Sexually Transmitted Diseases
